# Supplementary material for: Choline Hydrogen Dicarboxylate Ionic Liquids by X-ray Scattering, Vibrational Spectroscopy and Molecular Dynamics: H-Fumarate and H-Maleate and Their Conformations
Source: Molecules. 2020 Oct 28;25(21):4990. doi: 10.3390/molecules25214990 (PMC7663696; doi:10.3390/molecules25214990)
Supplement: Supplementary file 1 [file molecules-25-04990-s001.zip › Supplementary/Suppinfo.pdf]

EDXD technique

In EDXD experiments, about 0.3 ml of liquid were introduced in an amorphous quartz capillary (2 mm diameter), that was afterward sealed with a Teflon band and kept in dry atmosphere, just before the measurements. The EDXD (Energy Dispersive X-Ray Diffraction) technique is a variant of X-Ray Diffraction) technique is a variant of X-Ray diffraction that exploits the dependence of diffracted intensity on the energy of the radiation, according to the definition of the scattering variable for the Compton scattering between a photon and an electron:

$$q = \frac{4\pi sin\vartheta}{\lambda} \qquad 1)$$

This relation links the magnitude of the scattering variable (or momentum transfer) to the scattering angle (2ϑ) and to the wavelength of the incident radiation. Since the wavelength is related to the energy of the photon by the Planck equation E=hυ (h=Planck constant), we obtain

$$q = \frac{4\pi sin\vartheta}{hc} E \approx 1.014 E sin\vartheta \qquad 2)$$

Therefore, the whole spectrum of scattered wavevectors can be obtained either by varying the angle and keeping the radiation energy fixed (Angular Dispersive, ADXD, that uses a monochromatized beam) or by using a variable-wavelength energy beam (*i.e.* the“white”part of the radiation emitted by an X-Ray tube – Bremsstrahlung) and using a single angle, or a limited number of angles (3 to 4). The scattered intensity obtained at each angle undergoes a data treatment procedure, that normalizes the measured scattered intensity for absorption and subtracts self-scattering and the inelastic Compton incoherent scattering, yielding the total coherent structure function, which is also known as “reduced intensity”, I(q):

$$I(q) = I_{exp}(q) - \sum_{x=1}^{N} x_i f_i^2 - I_{Incoh} \qquad 3)$$

I(q) is the structural sensitive part of the recorded diffracted intensity I_{EXP}, as it depends on the sum of the interference contributions of the waves scattered by the atoms of the sample, and, ultimately, on their relative distances, as the atoms, through their electrons, are the particles taking part in the

scattering phenomenon (scatterers). The structure functions obtained from the different measurements (i. e. the 3-4 different angles) are finally joined to obtain a continuous spectrum in q, which in this study ranged from 0.5 to 24 $Å^{-1}$.

Several reviews on EDXD theory and applications, to which the reader is referred to, were published in the past[1,2]. A picture of the last prototype of EDXD instrument, with three detectors (three angles) in horizontal configuration is shown in Fig. S1.

From the Fourier Transform of I(Q), the total radial distribution function of the sample is obtained. This is a "real" space (or "direct" space) representation of the scattering, and is complementary to the I(q), as the same structural features may give a strong signature in I(Q) and a small one in the radial distribution, and vice versa. It is important to note that the radial distribution is a function of the (relative) distance, while the "reciprocal" space I(q) depends on momenta, that are inversely proportional to the distance (large distance = small momentum and *viceversa*). The functional form chosen for the total radial distribution functions shown in this work is the "differential"one (Diff (r)), defined as:

$$D(r) - 4\pi r^2 \rho_0 = Diff(r) = \frac{2r}{\pi}\int_0^\infty qI(q)M(q)rsin(q)dq \qquad 4)$$

In this expression, the non-structural term, due to the uniform radial distribution of particles that depends on the system density ρ, is left out. M(q) is a mathematical function used to reduce the truncation error of the Fourier Transform and to highlight the large q contributions, and is equal to $M(Q) = \frac{f^2N(0)}{f^2N(Q)}exp(-0.01q^2)$. The function I(q) is related to the partial radial distribution functions descriptive of the structure and obtainable from the simulations, according to Equation (5):

$$I(q) = \sum_{i=1}^{N}\sum_{j=1}^{N} x_i x_j f_i f_j 4\pi\rho_0 \int_0^\infty r^2 \left(g_{ij}(r) - 1\right)\frac{sin(qr)}{qr}dr \qquad 5)$$

In the Equations (3) and (5), x_i and x_j are the numerical concentrations of the species while f_i and f_j are their Q-dependent X-ray scattering factors and ρ_0 is the bulk number density. Equation (5) is the

link between experimental and model data, as the g(r)s can be calculated from molecular simulations. Comprehensive derivation of all the equations is described in[1].

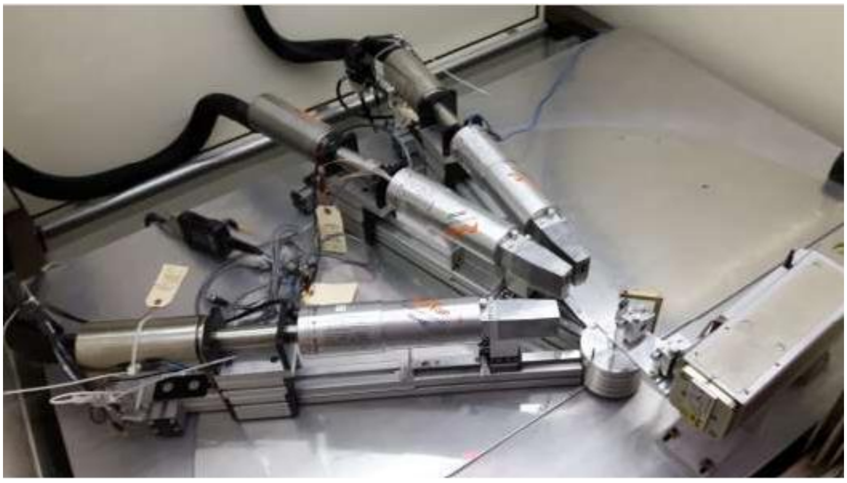

Fig. S1 – Photograph of the last prototype of EDXD diffractometer, with three detectors placed at three fixed angles
