# Supplementary material for: Choline Hydrogen Dicarboxylate Ionic Liquids by X-ray Scattering, Vibrational Spectroscopy and Molecular Dynamics: H-Fumarate and H-Maleate and Their Conformations
Source: Molecules. 2020 Oct 28;25(21):4990. doi: 10.3390/molecules25214990 (PMC7663696; doi:10.3390/molecules25214990)
Supplement: Supplementary file 1 [file molecules-25-04990-s001.zip › Supplementary/TableS1.pdf]

Table S1. Vibrational frequencies [cm^{-1}] calculated for the isolated and ion paired H-fumarate anion.

isolated anion		paired anion
Frequency	PED[a]	Frequency
30	τC1C2, 83	
108	τC2C3, δO1C1C2; 70, 17	
125	τC3C4, δC1C2C3, δC2C3C4; 41, 23, 16	
166	τC3C4, δC2C3C4, δC1C2C3; 34, 20, 15	
261	δC1C2C3, δC2C3C4, νC1C2 ; 24, 22, 17	304
388	δO1C1C2, δO4C4C3 ; 41, 13	394
455	δO4C4C3, δO1C1C2 ; 47, 11	561
550	τC4O4; 86	572
606	δO4'C4O4, δO1'C1O1 ; 56, 17	620
664	ωO1'C1O1, δO1'C1O1, δC4C3C2; 22, 21, 13	723
740	ωO4'C4O4, δO1'C1O1 ; 29, 17	681
781	ωO4'C4O4, ωO1'C1O1 ; 41, 28	802
882	γC2H, τC3H; 59, 11	936
910	νC1C2, νC4O4, δO1'C1O1; 39, 18, 14	935
974	νC3C4, δC1C2C3; 25, 17	970
994	γC3H, γC2H; 68, 20	1026
1107	νC4O4, δHO4C4, νC3C4, δHC3C4 ; 37, 23, 17, 11	1129
1245	δHC2C3, δHC3C4; δHO4C4; 42, 24, 20	1236
1284	δHC2C3, δHO4C4, δHC3C4; 34, 19, 15	1288
1343	δHC3C4, δHO4C4, νC2C3; 24, 20, 13	1356
1365	νC1O1', νC1O1, δO1'C1O1; 40, 32, 10	1387
1627	νC2C3, δHC3C4; 65, 10	1698
1662	νC1O1, νC1O1'; 53, 43	1615
1748	νC4O4'; 76	1780
3146	νC2H; 99	3181
3181	νC3H; 99	3200
3778	νO4H; 100	3768

[a] PED calculated for the isolated anion. ν, stretching; δ, bending; τ, torsion; ω, wagging, γ, out of plane bending.
