# Supplementary material for: Choline Hydrogen Dicarboxylate Ionic Liquids by X-ray Scattering, Vibrational Spectroscopy and Molecular Dynamics: H-Fumarate and H-Maleate and Their Conformations
Source: Molecules. 2020 Oct 28;25(21):4990. doi: 10.3390/molecules25214990 (PMC7663696; doi:10.3390/molecules25214990)
Supplement: Supplementary file 1 [file molecules-25-04990-s001.zip › Supplementary/TableS2.pdf]

Table S2. Vibrational frequencies [cm^{-1}] calculated for the isolated and ion paired H-maleate anion.

isolated anion		paired anion
Frequency	PED[a]	Frequency
56	τC1C2, τC3C4; 71, 18	
102	τC3C4, τC1C2; 57, 24	
279	τC2C3 ; 69	295
281	δC2C3C4, δC1C2C3, δO1C1O1'; 38, 15, 12	292
309	δC1C2C3, δO1C1C2, δO4C4C3; 35, 29, 17	317
402	δO4C4C3, δO1C1C2, νC2C3, νC1C2; 23, 20, 12, 12	423
569	δO4C4O4'; 60	610
592	τC3C4, ωO1C1O1', ωO4'C4O4, τC2C3 ; 29, 25, 23, 18	594
637	νC1C2, δO1C1O1', δO4C4C3 ; 36, 28, 23	641
777	νC3C4, δO1C1C2, δC2C3C4 ; 26, 25, 19	791
802	ωO4'C4O4, ωO1C1O1', τC2C3; 37, 35, 16	811
873	ωO1C1O1', γC2H, ωO4'C4O4, γC3H ; 28, 25, 24, 18	886
875	δO1'C1O1, νC3C4, δC2C3C4 ; 30, 12, 11	875
945	νC1C2, δC1C2C3, νC4O4, νC3C4, νC1O1; 20, 16, 15, 15,12	950
1012	γC3H, γC2H; 46, 44	1035
1197	νC4O4, νC1O1, νO4H; 27, 21, 14	1216
1210	τC4O4 ; 98	1039
1214	δHC2C3, δHC3C4; 42, 35	1218
1367	νO4H, νC1O1, νC1O1'; 27, 16	1367
1387	δHC3C4, δHC2C3; 44, 36	1415
1555	νC1O1', νO4H; 39, 25	1606
1639	δHO4C4, νC4O4', νC2C3; 35, 31, 14	1561
1655	νC2C3, δHO4C4; 58, 20	1668
1741	δHO4C4, νC4O4'; νC1O1'; 29, 21, 20	1772
1814	νO4H, ν C4O4', ν C1O1; 40, 17, 13	2805
3128	νC2H, νC3H; 60, 40	3154
3154	νC3H, νC2H; 60, 40	3175

[a] PED calculated for the isolated anion. ν, stretching; δ, bending; τ, torsion; ω, wagging, γ, out of plane bending.
